# Co-Infection between Dengue Virus and SARS-CoV-2 in Cali, Colombia

**DOI:** 10.4269/ajtmh.22-0717

**Published:** 2023-08-14

**Authors:** Olga Lucia Agudelo-Rojas, David Esteban Rebellón-Sánchez, Julio Llanos Torres, Isabel Lucia Zapata-Vásquez, Sarita Rodríguez, Sebastián Robles-Castillo, Alejandro Tejada Vega, Luis Gabriel Parra-Lara, Fernando Rosso

**Affiliations:** ^1^ Centro de Investigaciones Clínicas, Fundación Valle del Lili, Cali, Colombia;; ^2^ Facultad de Ciencias de la Salud, Universidad Icesi, Cali, Colombia;; ^3^ Departamento de Salud Pública y Medicina Comunitaria, Facultad de Ciencias de la Salud, Cali, Colombia;; ^4^ Servicio de Enfermedades Infecciosas, Departamento de Medicina Interna, Fundación Valle del Lili, Cali, Colombia;; ^5^ Departamento de Ciencias Clínicas, Universidad Icesi, Cali, Colombia

## Abstract

The co-occurrence of COVID-19 with endemic diseases is a public health concern that may affect patient prognosis and outcomes. The objective of this study was to describe the clinical characteristics of patients with dengue virus (DENV) and SARS-CoV-2 co-infections and compare their outcomes against those of COVID-19 patients without dengue. A cross-sectional study was conducted in patients with SARS-CoV-2 infection who attended a single center in Cali, Colombia, from March 2020 to March 2021. All patients who were tested by both real-time polymerase chain reaction for SARS-CoV-2 and IgM/NS1 for DENV were included. Dengue was diagnosed as having either an IgM- or an NS1- positive test. A total of 90 patients were included (72 with COVID-19 only and 18 with co-infection). Patients with co-infection had more dyspnea (61.1% versus 22.2%; *P* = 0.003) as well as higher oxygen desaturation (53.3% versus 13.4%; *P* = 0.002) and neutrophil-to-lymphocyte ratio (5.59 versus 3.84; *P* = 0.038) than patients with COVID-19 alone. The proportion of patients classified with moderate to severe COVID-19 was higher in the co-infection group (88.3% versus 47.8%; *P* = 0.002). Also, co-infection was associated with an increased need for mechanical ventilation (*P* = 0.06), intensive care unit (ICU) initial management (*P* = 0.02), and ICU admission during hospitalization (*P* = 0.04) compared with COVID-19 only. The ICU mortality rate was 66.6% in patients with co-infection versus 29.4% in patients infected with only SARS-CoV-2 (*P* < 0.05). The possibility of DENV and SARS-CoV2 co-infection occurred in the convergence of both epidemic waves. Co-infection was associated with worse clinical outcomes and higher mortality in ICU-admitted patients than in patients with the COVID-19 only.

## INTRODUCTION

The COVID-19 pandemic has generated a high burden of morbidity and mortality globally. The co-occurrence of COVID-19 with endemic diseases is a public health concern that may affect patient prognosis and outcomes.^1^ According to the Instituto Nacional de Salud, the incidence of dengue in Colombia was 255.97 cases per 100,000 inhabitants, with 78,979 cases reported in 2020.^2^ Surveillance of co-circulation of SARS-CoV-2 and dengue virus (DENV) is important for public health because of the high transmissibility of the viruses and the ability of both to produce potentially fatal outcomes, especially in patients with comorbidities.^3^

Differentiating between these two entities is very challenging because of the remarkable similarities in pathophysiology, clinical manifestations, and blood work findings of the diseases.^4^^,^^5^ Moreover, some reports that COVID-19 infection generated false-positive results for DENV infection have further complicated discrimination of these two entities. This finding could also cause delays and errors in the diagnosis of COVID-19 infection, leading to further spread of the virus and dissemination of the disease and directly impacting the morbidity/mortality of the population.^6^^,^^7^ Faced with this challenge, we conducted a previous study indicating that during the first week of symptoms, absolute neutrophil count, neutrophil-to-lymphocyte ratio (NLR), and platelet count could help guide the initial differentiation between dengue and COVID-19.^8^

In addition, when these two entities are considered as differential diagnoses, there is always a possibility of co-infection by SARS-CoV-2 and DENV, especially in territories where both viruses are endemic or hyperendemic. Co-circulating and nonspecific presentations increase the chance of misdiagnosis, generate a great demand for healthcare services, and could also worsen patients’ prognoses. The objective of this study was to describe the clinical characteristics of patients with co-infection and compare their outcomes with those of patients with COVID-19 alone in a single center in Cali, Colombia.

## MATERIALS AND METHODS

### Study design.

A cross-sectional study was conducted at Fundación Valle del Lili, a nonprofit teaching hospital in Cali, Columbia, that serves as a referral center for the southwest region of the country.

From the total number of adult patients with COVID-19 seen at the hospital in March 2020–March 2021, all patients who were tested by both real-time polymerase chain reaction (RT-PCR) for SARS-CoV-2 and by IgM or NS1 for DENV were selected (Supplemental Material). Patients were included regardless of sex or clinical condition. Patients were then classified in two groups: (1) patients with SARS-CoV-2 infection only and (2) patients coinfected with DENV and SARS-CoV-2. It is worth mentioning that patients presenting to our center with suspected or confirmed cases of COVID-19 were not routinely tested for dengue as part of their clinical care.

In Colombia, there were 48,214 cases of dengue fever in the period analyzed, of which 605 cases were severe dengue and 44 patients died.^9^ Meanwhile, in the same period there were 2,397,731 cases of COVID-19, and 63,422 patients died.^10^

### Ethics considerations.

This study was approved by the Institutional Review Board (IRB) – Comité de Ética en Investigación Biomédica at Fundación Valle del Lili (IRB/EC No. 1566) and was conducted following the Declaration of Helsinki and recommendations of the Council for International Organizations of Medical Sciences. Patient consent was not required because this study was performed retrospectively.

### Variables and data collection.

Demographic, clinical (diagnosis, sign and symptoms, initial management), and clinical outcome variables were collected. We identified COVID-19 patients from the Institutional COVID-19 Registry and dengue patients from the microbiology laboratory database and databases of dengue studies conducted in the hospital. The COVID-19 Registry is an institutional initiative approved by the Institutional Ethics Committee that was developed at the beginning of the pandemic, in which the clinical, laboratory, and microbiological data of all patients with COVID-19 treated at Fundación Valle de Lili since the beginning of the pandemic were included. This information was authorized for use in research, subject to ethical approval of a new protocol reviewed by the IRB protecting the identity of the participants and complying with national and international regulations on good clinical practices and health research.

COVID-19 severity was defined using WHO severity definitions based on clinical indicators.^11^ These definitions include (1) asymptomatic: a person infected with SARS-CoV-2 who does not develop symptoms; (2) mild: symptomatic patients meeting the case definition for COVID-19 without evidence of viral pneumonia or hypoxia; (3) moderate: patient with clinical signs of pneumonia (fever, cough, dyspnea, fast breathing) but no signs of severe pneumonia, including pulse oxygen saturation (SpO_2_) ≥ 90% on room air; (4) severe: patient with clinical signs of pneumonia (fever, cough, and dyspnea) plus one of the following: respiratory rate > 30 breaths/minute, severe respiratory distress or SpO_2_ < 90% on room air; (5) critical: patient with criteria for acute respiratory distress syndrome (ARDS), sepsis, septic shock, acute thrombosis, or other conditions that would normally require the provision of life-sustaining therapies such as mechanical ventilation (invasive or noninvasive) or vasopressor therapy.

### Diagnosis of SARS-CoV-2 and DENV infections.

SARS-CoV-2 infections were diagnosed with nasopharyngeal swabs using the CDC 2019-nCoV RT-PCR Diagnostic Panel protocol (CDC, Atlanta, GA), VIASURE SARS-CoV-2 RT-PCR Detection Kit (Certest Biotec S.L., Zaragoza, Spain), Allplex 2019-nCoV Assay (Seegene Inc, Seoul, South Korea), or AccuPower SARS-CoV-2 Multiplex RT-PCR Kit (Bioneer Corporation, Daedeok-gu, South Korea). Dengue cases were diagnosed through blood samples using the SD BIOLINE Dengue DUO NS1 Ag + Ab Combo test (Alere Inc., Waltham, MA). None of the participants classified as having dengue had confirmation of the virus by molecular detection because of the unavailability of the test in Colombia in routine clinical practice.

### Statistical analyses.

A descriptive analysis was performed. The Q-Q plot and Shapiro-Wilk test were used to assess the normality of quantitative variables. All quantitative variables had a non-normal distribution and were summarized with the median and interquartile range. The SARS-CoV-2 group and the DENV and SARS-CoV-2 co-infection group were compared using the Mann-Whitney *U* test. Qualitative variables are shown as frequencies and percentages and were compared using the χ^2^ test or Fisher’s exact test. A 5% significance level was applied. All analyses were performed using STATA (version 17.0, StataCorp LP, College Station, TX).

## RESULTS

A total of 90 patients were included in the study, of whom 72 had SARS-CoV-2 infection and 18 had co-infection. All 90 participants had positive IgG tests for DENV, confirming the high endemicity of the disease in our population. The 72 patients with exclusive SARS-CoV-2 infections were dengue-negative (none had IgM or NS1 antigen positive for DENV). All 18 participants with co-infections were dengue-IgM positive and one was also NS1-antigen positive. Most patients were males (65.6%), with a median symptom duration of 5 days. The most common symptom in both groups was fever (75% in the SARS-CoV-2 group versus 72.2% in the co-infected group). Dyspnea (*P* = 0.003) and oxygen desaturation (*P =* 0.002) were more common in co-infected patients. None of the patients in the cohort had received the COVID-19 vaccine because it was not yet available in Colombia during the study period. In addition, 27.8% (*n* = 5) of the co-infected group had severe dengue, 21.8% (*n* = 5) had dengue with alarm signs, and 44.4% (*n* = 8) had dengue without alarm signs. No differences were found after comparison of demographic characteristics and medical history. Table 1 shows the demographic and clinical characteristics of the patients.

**Table 1:** Demographic and clinical characteristics of the patients included (*N* = 90)

Characteristics	Overall,	SARS-CoV-2 infection only	Coinfection	*P* value
	(*N* = 90)	(*n* = 72)	(*n* = 18)	
Demographic				
Median age (IQR), year	50 (37–59)	48 (18–83)	50 (24–78)	0.670
Male sex, *n* (%)	59 (65.5)	46 (63.9)	13 (72.2)	0.698
Medical history, *n* (%)				
Overweight/Obesity*	49 (59.1)	39 (58.2)	10 (62.5)	0.976
Hypertension	24 (26.6)	17 (23.6)	7 (38.9)	0.311
Diabetes	14 (15.5)	12 (16.7)	2 (11.1)	0.827
Chronic renal disease	10 (11.1)	8 (11.1)	2 (11.1)	1
Lung disease	6 (6.6)	6 (8.3)	–	0.460
Transplant	5 (5.5)	5 (6.9)	–	0.565
Cancer	5 (5.5)	4 (5.6)	1 (5.9)	1
Heart disease	4 (4.4)	2 (2.8)	2 (11.1)	0.371
Chronic liver disease	2 (2.2)	2 (2.8)	–	1
Autoimmune disease	3 (3.3)	2 (2.8)	1 (5.6)	1
Cerebrovascular disease	1 (1.1)	–	1 (5.6)	0.451
Median days of symptoms (IQR)	5 (3–8)	5 (3–8)	5.5 (3.2–9.5)	0.274
Signs and symptoms, *n* (%)				
Fever	67 (74.4)	54 (75)	13 (72.2)	1
Fatigue	43 (47.7)	32 (44.4)	11 (61.1)	0.316
Myalgias	39 (43.3)	35 (48.6)	4 (22.2)	0.079
Cough	39 (43.3)	32 (44.4)	7 (38.9)	0.873
Headache	37 (41.1)	32 (44.4)	5 (27.8)	0.309
Arthralgias	29 (32.2)	26 (36.1)	3 (16.7)	0.195
Dyspnea	27 (30)	16 (22.2)	11 (61.1)	0.003
Odynophagia	20 (22.2)	17 (23.6)	3 (16.7)	0.751
Oxygen desaturation†	17 (18.8)	9 (13.4)	8 (53.3)	0.002
Rhinorrhea/Nasal congestion	15 (16.6)	11 (15.3)	4 (22.2)	0.724
Diarrhea	14 (15.5)	11 (15.3)	3 (16.7)	1
Abdominal pain	11 (12.2)	8 (11.1)	3 (16.7)	0.809
Vomiting	8 (8.8)	6 (8.3)	2 (11.1)	1
Nausea	7 (7.7)	5 (6.9)	2 (11.1)	0.922
Expectoration	6 (6.6)	4 (5.6)	2 (11.1)	0.751
Anorexia	5 (5.5)	5 (6.9)	–	0.565
Taste alteration	5 (5.5)	3 (4.2)	2 (11.1)	0.565
Chest pain	3 (3.3)	2 (2.8)	1 (5.6)	1
Dysphagia	2 (2.2)	2 (2.8)	–	1

IQR = interquartile range.

* No co-infection *n* = 67, co-infection *n* = 16.

† No co-infection *n* = 67, co-infection *n* = 15.

In addition, when clinical severity was compared by age, it was observed that older patients were more likely to have moderate or severe COVID-19 than younger patients (70.2% of patients aged 50 years or older versus 34.9% in patients younger than 50 years; *P* = 0.008). When only the co-infection group was analyzed, 100% (*n* = 10) of patients aged 50 years or older developed moderate or severe COVID-19, whereas in patients younger than 50 years only 62.5% developed moderate or severe COVID-19 (*P =* 0.17). In contrast, when the severity of dengue in co-infected participants was analyzed, it was found that participants younger than 50 years were more likely to develop dengue with alarm signs or severe dengue than patients aged 50 years and older (62.5% versus 50%). However, these differences did not reach statistical significance (*P =* 0.66).

**Figure 1.**
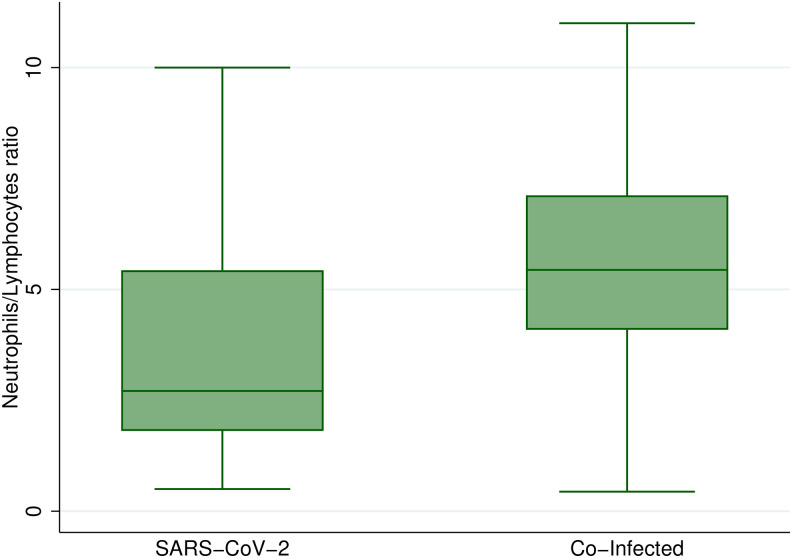
Comparison of neutrophil-to-lymphocyte ratio. Co-infected patients exhibited significantly higher neutrophil/lymphocyte ratios than patients with only SARS-CoV-2 infection (median 5.44 interquartile range [IQR: 4.09–7.12] vs. median 2.71 [IQR: 1.81–5.43]; *P* = 0.03).

A higher NLR was observed in the co-infected group (*P* = 0.038) (Figure 1). Other laboratory blood tests, such as platelet count, total bilirubin, and transaminase levels, showed similar values between the groups (Supplemental Material).

**Figure 2.**
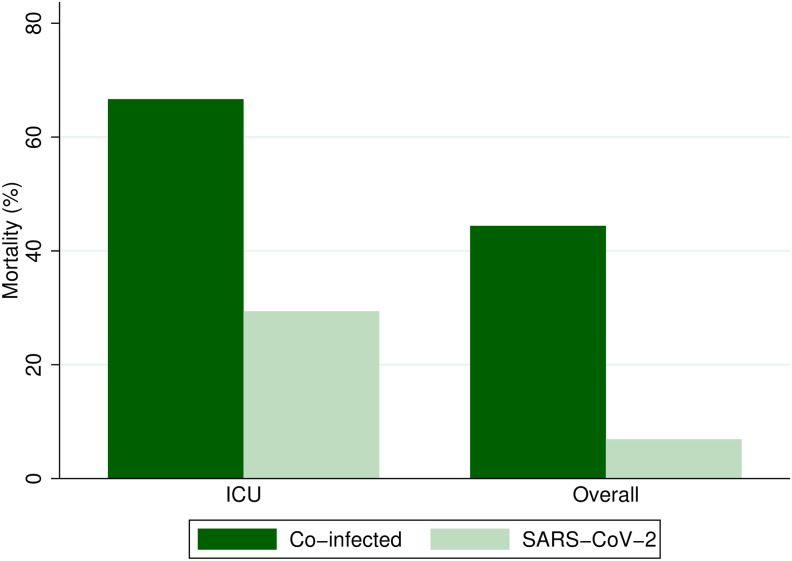
Comparison of overall and intensive care unit (ICU) mortality rates according to infection group. Patients with dengue virus–SARS-CoV-2 co-infection exhibited higher overall mortality rates than patients with SARS-CoV-2 infection. After adjustment for severity and focusing solely on patients with severe symptoms requiring ICU management, a statistically significant difference persisted, with a greater proportion of deaths observed in the co-infection group.

Most of the patients with co-infection presented with moderate and severe COVID-19 (*P* = 0.002) compared with those with COVID-19 only. A higher proportion of patients with co-infection required intensive care unit (ICU) management at admission (53.3% versus 20.6%; *P* = 0.02), initial oxygen support (73.3% versus 55.9%; *P =* 0.20), mechanical ventilation (60% versus 32.4%; *P =* 0.06), and intensive care unit management during their hospitalization (80% versus 50%; *P* = 0.04). In contrast, a higher proportion of patients with COVID-19 alone required outpatient treatment compared with those in the co-infection group (52.8% versus 16.7%; *P* = 0.002) (Table 2). Likewise, higher overall and ICU mortality rates were observed in patients with co-infection compared with the COVID-19 alone group (overall mortality: 44.4% versus 6.9%, *P =* 0.001; ICU mortality: 66.7% versus 29.4%, *P =* 0.04) (Figure 2). In addition, all co-infected patients with severe COVID-19 had computed tomography findings compatible with ARDS, and none of them had findings of third space involvement. Of the total number of patients with co-infection, only one patient had third space involvement, which was clinically classified as moderate COVID-19.

**Table 2:** COVID-19 severity, therapeutic requirements, and clinical outcomes in inpatients (*N* = 49)

Characteristics	Overall	SARS-CoV-2 infection only	Coinfection	*P* value
	(*N* = 49)	(*n* = 34)	(*n* = 15)	
COVID-19 severity at admission, *n* (%)				
Asymptomatic	1 (2.04)	1 (2.94)	–	0.23
Mild	8 (16.3)	6 (17.6)	2 (13.3)	
Moderate	24 (49)	19 (55.9)	5 (33.3)	
Severe	16 (32.7)	8 (23.5)	8 (53.3)	
Initial admission management, *n* (%)				
Hospitalization	34 (69.4)	27 (79.4)	7 (46.7)	0.04
ICU	15 (30.6)	7 (20.6)	8 (53.3)	
Initial oxygen support requirement, *n* (%)	30 (61.2)	19 (55.9)	11 (73.3)	0.20
Mechanical ventilation, *n* (%)	20 (40.8)	11 (32.4)	9 (60)	0.06
Final management during the hospitalization, *n* (%)				
Hospitalization	20 (440.8)	17 (50)	3 (20)	0.04
ICU	29 (59.2)	17 (50)	12 (80)	
ICU mortality, *n* (%)	13 (44.8)	5 (29.4)	8 (66.7)	0.05
Vital status at discharge, *n* (%)				
Alive	36 (73.5)	29 (85.3)	7 (46.7)	0.008
Deceased	13 (26.5)	5 (14.7)	8 (53.3)	

ICU = intensive care unit.

Nonsignificant differences were found in mortality by sex between those who died (*n* = 13) and those who survived (*n* = 77) (18.6% in men versus 6.5% in women; *P =* 0.20). There was a higher proportion of deaths in those aged 50 years or older than in those younger (21.3% versus 6.9%; *P =* 0.05). In addition, the proportion of patients with co-infection was higher in the mortality group than in those who survived (61.5% versus 13%; *P <* 0.001). Thus, 20% (*n* = 2/8) of patients with co-infection younger than 50 years died versus 60% (*n* = 6/10) of those 50 years and older. Among patients with co-infection, 100% (*n* = 5) of the participants classified as having severe dengue, 20% (*n* = 1) of those with dengue with alarm signs, and 25% (*n* = 2) of those with dengue without alarm signs died. Of the patients with only SARS-CoV-2 infection, 50% of those classified as having severe COVID-19 (*n* = 8), 12.5% of those with moderate COVID-19 (*n* = 4), and 2.9% of those with mild COVID-19 died. Table 3 shows a comparative analysis by mortality group.

**Table 3:** Clinical characteristics of deceased patients (*N* = 13)

Characteristic	Patient												
	1	2	3	4	5	6	7	8	9	10	11	12	13
Age, years	59	58	78	44	51	57	49	75	77	64	83	42	70
Sex	M	M	M	M	M	M	M	F	M	M	M	M	M
Patient classification	Co- infected	Co- infected	Co- infected	Co- infected	Co- infected	Co- infected	Co- infected	Co- infected	COVID-19	COVID-19	COVID-19	COVID-19	COVID-19
Comorbidity	HTN, T2DM, OO	HTN, OO	HTN	OO	HTN, T2DM, OO	HTN	HTN	CVD, OO	HTN	T2DM	T2DM	Cancer	None
COVID-19 severity at admission	Severe	Severe	Mild	Severe	Severe	Severe	Mild	Mild	Severe	Severe	Mild	Mild	Severe
WHO dengue classification	DNWS	DNWS	SD	SD	SD	SD	SD	DWWS	–	–	–	–	–
NEWS	6	9	1	15	10	13	9	5	13	7	2	5	9
Length of hospital stay, days	64	31	56	7	17	7	25	35	3	42	30	34	9
Length of ICU stay, days	63	30	51	6	16	7	23	19	2	31	22	27	9
Time of vasopressor support requirement, days	0	5	15	7	15	7	20	10	2	15	0	0	4
IMV requirement	No	Yes	Yes	Yes	Yes	Yes	Yes	Yes	Yes	Yes	Yes	Yes	Yes
Time of IMV, days	0	31	51	7	16	7	22	19	2	25	22	12	7

CVD = cerebrovascular disease; DNWS = dengue without warning signs; DWWS = dengue with warning signs; F = female; HTN = hypertension; ICU = intensive care unit; IMV = invasive mechanical ventilation; M = male; NEWS = National Early Warning Score; OO = overweight or obesity; SD = severe dengue; T2DM = type 2 diabetes mellitus.

## DISCUSSION

Dengue fever is an endemic disease in Latin America,^12^ and the advent of the COVID-19 pandemic showed overlapping symptoms of COVID-19 and dengue fever. This represents a diagnostic challenge for health care workers as these diseases share symptoms and blood work findings, making it difficult to differentiate between them.

Cases have even been described in which SARS-CoV-2 infection generated false-positive DENV tests.^4^ However, the literature is limited, and most data refer to small cohort studies conducted in Southeast Asian countries. A major limitation in low-income countries is that despite being endemic areas for dengue infections, confirmatory molecular tests for DENV are not available in routine clinical practice. Currently, dengue diagnosis is based on clinical manifestations and antibody or NS1 positivity, which can lead to misdiagnosis. A greater public health effort is required to implement molecular diagnostic tests in endemic countries to ensure better access to diagnostic tools in clinical practice and to improve epidemiological surveillance through, for example, real-time monitoring of circulating variants.

In Latin America, there have been reports of SARS-CoV-2 and DENV co-infection. The first case was reported in Brazil, a 58-year-old woman with no medical history who presented with mild cases of both viral infections with a favorable evolution and no complications. In a systematic review published in December 2022 on SARS-CoV-2 and DENV co-infection in Latin America, only four descriptive studies with at least 10 patients were identified.^13^

In Argentina, a retrospective study with 13 patients with co-infection showed that most patients presented with mild COVID-19 (12/13, 92%), none of whom had severe dengue or required ICU management.^14^ In contrast, in another retrospective study in Peru that included 50 patients with co-infection, 6% (3/50) developed severe dengue and were treated in the ICU. The case fatality rate was 28% (14/50), and all patients with severe dengue died; however, no comparisons were made with patients with only COVID-19 infection.^15^ In Brazil, a retrospective cohort study of 43 cases of SARS-CoV-2 and DENV co-infection reported that active dengue worsened pulmonary function in patients with COVID-19, a finding comparable to ours.^16^ Another retrospective study conducted in midwestern Brazil found 13 SARS-CoV-2 and DENV co-infections; 30.8% of patients were hospitalized, none of them died, and clinical improvement was achieved in all patients after a maximum of 21 days.^17^

Given the overlapping clinical features and blood work findings, clinical suspicion is important when COVID-19 and dengue diagnoses are made. According to our results, epidemiological criteria are not sufficient to differentiate COVID-19 from dengue. In this study, the two diseases shared clinical manifestations such as fever, cough, fatigue, myalgias, and arthralgias. Although respiratory symptoms are one of the characteristics that can guide the clinical diagnosis of COVID-19,^18^ patients with co-infection had a higher risk of developing severe respiratory symptoms such as dyspnea and desaturation.

In addition, it is important to mention that patients with severe dengue are mainly young patients. Our previous report noted that complications resulting from severe dengue were seen in younger patients. In contrast, older adults may present with severe cases and complications due to COVID-19.^8^ Consistent with our previous observations, in this study we found that co-infected patient 50 years or older tended to have more severe clinical manifestations of COVID-19, whereas those younger than 50 years tended to have greater dengue severity. Moreover, mortality was significantly higher in patients aged 50 years and older than in younger patients. Therefore, age appears to be an important determinant of clinical severity in patients with co-infection.

Among the biochemical alterations found in patients with SARS-CoV-2 and DENV co-infection, lymphopenia was a common finding, which has been described in other studies.^5^ In our study, patients with co-infection presented with lymphopenia, but it did not represent a criterion to differentiate these two diseases. Moreover, NLR has been reported as a marker of severity and poor prognosis in patients with COVID-19,^19^^,^^20^ and our previous research found that NLR was useful for differentiating between dengue and COVID-19, being higher in patients with only COVID-19.^8^ However, this new study found that NLR in the co-infection group was significantly higher, reflecting potential utility as an easy and accessible serum biomarker for co-infection.

There is still no consensus in the literature regarding the clinical outcomes of patients with co-infection because previous studies showed contradictory results. Although co-infection has been reported to be associated with mild disease and favorable prognosis in some studies, in other studies it has been associated with severe disease and increased mortality.^14^^,^^15^^,^^21^ However, it has been reported that both infections involve different proinflammatory cascades such as CCL4 and AhR, which can perpetuate and exacerbate the clinical presentation of SARS-CoV-2 and DENV co-infection. A high concentration of CCL4 has been identified in secretions of patients with severe COVID-19, although its expression has also been found in dengue-infected dendrites, promoting vasodilatation and endothelial dysfunction in both diseases.^22^ This suggests that co-infection results in more severe disease and therefore greater morbidity and mortality, which is consistent with our results. Our results show that co-infected patients had not only more severe disease at admission but also a higher need for initial oxygen support, mechanical ventilation, ICU management, and overall and ICU mortality rates than patients with COVID-19 only.

### Limitations.

Our study has certain limitations, and results must be interpreted with respect to the study design. First, our hospital is a high-complexity center, so patients treated here have more comorbidities, advanced conditions, and complications; therefore, a selection bias may have been introduced. Second, data were obtained directly from medical records and secondary databases, which could lead to information bias regarding symptoms and clinical presentation of the patients included. Third, all laboratory test results used in the analysis were taken at the moment of admission to the hospital. Finally, in Colombia, according to clinical practice guidelines, the diagnosis of dengue is determined by serological tests, which have lower performance than the RT-PCR method, thus increasing the risk of false results during diagnosis.^23^ In the context of high COVID-19 transmissibility, it is expected that IgM for DENV could be falsely positive in some patients classified as co-infected who were actually mono-infected.

On the other hand, the study was developed prior to the wide availability of vaccination, and none of the patients in the cohort had received the COVID-19 vaccine; therefore, our results may have been different if this intervention had been considered. Another limitation is that within the included patients, we had more severe cases of dengue in relation to a smaller number of patients with severe COVID-19, which may have led to selection bias. For this reason, we decided to compare overall and ICU mortality rates, as patients admitted to the ICU had similar disease severity and were therefore more comparable. When this subanalysis was performed, patients with co-infection continued to present with higher mortality, which improved consistency in our findings.

## CONCLUSION

The possibility of co-infection with DENV and SARS-CoV-2 could occur in the convergence of epidemic waves of both infections. Clinical manifestations, exposure history, and blood work findings should be considered important tools for differential diagnosis. According to our results, patients with co-infection presented with severe respiratory symptoms and an elevated NLR. Co-infection was associated with worse clinical outcomes and high mortality in ICU-admitted patients. In our experience, in young patients with co-infection, complications tended to be more related to dengue symptoms, whereas in older patients, complications tended to be more related to COVID-19 symptoms.

## Supplemental Materials


Supplemental materials
