# Supplementary material for: Co-Infection between Dengue Virus and SARS-CoV-2 in Cali, Colombia
Source: Am J Trop Med Hyg. 2023 Aug 14;109(3):536–41. doi: 10.4269/ajtmh.22-0717 (PMC10484269; doi:10.4269/ajtmh.22-0717)
Supplement: Supplementary file 1 [file tpmd220717.SD1.pdf]

SUPPLEMENTARY FILE

Figure S1. Flowchart of the study.

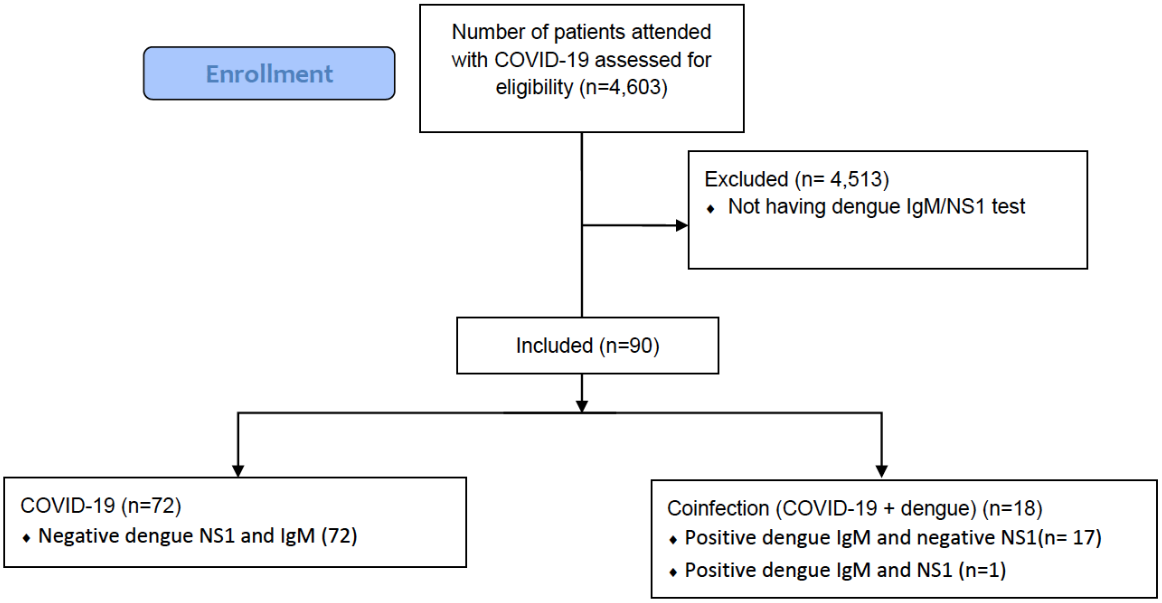

Table S1. Laboratory findings in the cohort.

Laboratory tests	COVID-19 only, n=72	Coinfection, n=18	p-value
**Median laboratory parameters at admission (IQR)**			
Leukocytes count - /µL	6,015 (4,942-7,052)	6,950 (5,260-10,720)	0.153
Neutrophils count - /µL	4,075 (3,255-5,747)	5,060 (3,210-9,000)	0.110
Lymphocytes count - /µL	1,180 (795-1,520)	740 (650-1,230)	0.117
NLR	3.84 (2.37-6.16)	5.59 (3.87-14.48)	0.038
Hematocrit - %	41.60 (38.93-45.75)	41.30 (37.70-45.70)	0.564
Platelets count – x10^3/ µL	184 (142.75-238)	5 (138-205)	0,660
Total bilirubin – mg/dL	0.42 (0.27-0.74	0.44 (0.38-0.86)	0.369
AST – U/L	32.6 (23.6-49.9)	55.7 (22.4-78.6)	0.446
ALT – U/L	35.8 (23.1-67.3)	62.3 (34.2-76.5)	0.114

Table S2. Comparative analysis by mortality group.

Characteristic	Death, n=13	Alive, n=77	p-value
Median age (IQR) – yr	59 (51-75)	48 (35-59)	0.003*
<50 yr, n (%)	3 (23.08)	40 (51.95)	0.054‡
≥50 yr, n (%)	10 (76.92)	37 (48.05)	
Sex, n (%)			
Male	2 (15.38)	29 (37.66)	0.2‡
Female	11 (84.62)	48 (62.34)	
Median length of symptoms - days, n (%)	5 (3-8)	5 (3-8)	0.5*
Classification, n (%)			
Co-infected	8 (61.54)	10 (12.99)	<0.001†
COVID-19	5 (38.46)	67 (87.01)	
WHO dengue classification, n (%)			
Dengue without signs	2 (25)	6 (60)	0.015‡
Dengue with warning signs	1(12.50)	4 (40)	
Severe dengue	5 (62.50)	-	
COVID-19 severity, n (%)			
Asymptomatic	0 (0)	7 (9.09)	<0.001‡
Mild	1 (7.69)	34 (44.16)	
Moderate	8 (61.54)	8 (10.39)	
Severe	4 (30.77)	28 (36.36)	
Median SOFA score at admission (IQR)	6 (5, 7)	2 (1, 3.25)	0.007*
Median APACHE II at admission (IQR)	18 (12-23)	6 (4-11)	0.017*
Median NEWS score at admission (IQR)	9.0 (5-10)	2 (1-5)	<0.001*
Median time of vasopressor support requirements (IQR) - days	8.5 (5.5-15)	0	0.001*
Median length of ICU stay (IQR) – days	22 (9-30)	7 (4-16)	0.014*
Median length of hospital stay (IQR) – days	30 (9-35)	1 (0-6)	<0.001*
Oxygen therapy required at admission, n (%)			
None	3 (23.08)	55 (73.33)	<0.001‡
Nasal cannula	1 (7.69)	13 (17.33)	
High flow nasal cannula	2 (15.38)	2 (2.67)	
Non-rebreather mask	3 (23.08)	3 (4)	
IMV	4 (30.77)	2 (2.67)	
IMV requirement during hospitalization, n (%)	12 (92.31)	8 (10.39)	<0.001‡
Median time of IMV requirement (IQR) - days	18 (7-23)	11 (8-14)	0.3*

*Wilcoxon rank sum test

‡Fisher's exact test

†Pearson's Chi-squared test

APACHE: Acute Physiology and Chronic Health disease Classification System; IMV: invasive mechanical ventilation; IQR: interquartile range; NEWS: National Early Warning Score; SOFA: Sepsis related Organ Failure Assessment; WHO: World Health Organization
